# Evaluation of Extended-Release Eprinomectin Injectable and Doramectin Injectable with the Inclusion of Refugia on Performance Outcomes and Fecal Parameters in Stocker Cattle

**DOI:** 10.3390/vetsci12040352

**Published:** 2025-04-09

**Authors:** Daniel B. Cummings, J. Oliver Irons, Jennifer Surotchak, David Renter

**Affiliations:** 1 Heritage Veterinary Partners, Madisonville, TN 37354, USA; 2 Ironsides Animal Health, PLLC, Lewisburg, WV 24901, USA; oliver@ironsidesgroup.com (J.O.I.); jennifer@ironsidesgroup.com (J.S.); 3 Center for Outcomes Research and Epidemiology, Department of Diagnostic Medicine/Pathobiology, College of Veterinary Medicine, Kansas State University, Manhattan, KS 66506, USA; drenter@vet.ksu.edu

**Keywords:** stocker cattle, anthelmintics, refugia, parasite control

## Abstract

Evaluating different tactics to mitigate the effects of gastrointestinal nematode infection in growing stocker cattle is essential to better understand opportunities to optimize cattle health and performance. Due to the potential development of anthelmintic resistance, parasitologists and industry stakeholders have proposed maintaining refugia in cattle populations and combination treatment as tactics to delay anthelmintic resistance yet limited large-scale field data are available for practitioners to make evidence-based decisions. The objective of this experiment was to compare the effects of extended-release eprinomectin injectable and doramectin injectable on growth and fecal shedding of parasites in stocker calves grazing with non-treated (refugia) cohorts. Steers (*n* = 995; 243.38 kg) were randomized to one of two experimental treatment groups on Day 0, extended-release eprinomectin (ERE) or doramectin injectable (DOR). A subset of animals (*n* = 47) was selectively not treated with an anthelmintic to maintain refugia (REF). Individual body weights were recorded on days 0, 105, and 130 to calculate average daily gain and overall body weight gain during each of those time periods. Fecal samples were collected per rectum from approximately 10% of the same animals in each group on days 0, 105, and 130. Mean fecal egg count was significantly lower in the 10% of animals tested in the ERE group on days 105 and 130 when compared to 10% of the animals in the DOR group (Day 105—ERE: 46.45 eggs per gram, DOR: 155.30 eggs per gram, *p* < 0.01; Day 130—ERE: 9.65 eggs per gram, DOR: 22.51 eggs per gram, *p* = 0.02). From day 0 to 105, the mean average daily gain in the ERE group was 0.87 kg/day, which tended (*p* = 0.055) to be higher than the mean for the DOR group, 0.845 kg/day.

## Simple Summary

Gastrointestinal nematodes may infect young, growing calves resulting in significant performance losses. Cattle entering stocker operations known for continuous grazing patterns may be at increased risk for the development of anthelmintic resistance. As such, tactics to maximize performance and mitigate the risk of anthelmintic resistance including the maintenance of refugia and/or combination treatment must be explored in these systems. The purpose of this experiment was to compare the effects on performance and parasite egg shedding of extended-release eprinomectin (ERE) and doramectin injectable (DOR), administered to cattle grazed with untreated cohorts serving as a source of refugia (REF). Steers procured by a commercial stocker operation were administered either ERE or DOR. A subset of animals (REF) was selectively not treated and commingled with the experimental treatment groups for the duration of the grazing period. A tendency for improved average daily gain was observed in the ERE group when compared to the DOR group. In addition, average fecal egg counts were lower at two time points in the 10% of calves sampled in the ERE group in comparison to the 10% sampled in the DOR group.

## 1. Introduction

Stocker cattle enterprises are uniquely positioned between the cow-calf and feeder phase of production, serving an important role in supplying quality feeder cattle downstream to feedlot operations [1]. Stocker cattle enterprises frequently rely on pastureland to prepare the young, growing beef calf procured from local marketing channels to the feeder segment. Due to continuous animal throughput, continuous grazing of permanent pastures, and changing climate conditions, parasitologists and veterinarians have suggested the impacts of gastrointestinal nematodes (GINs) are of greater negative magnitude in stocker operations compared to cow-calf and feedlot systems, particularly in the Southeast and Atlantic regions of the United States (US) [2,3]. Therefore, stakeholders must critically evaluate traditional parasite control programs to account for short- and long-term production goals, focusing on optimizing cattle health and performance.

Gastrointestinal nematodes frequently identified as having the greatest impact in stocker cattle include *Ostertagia ostertagi*, *Haemonchus placei*, *Cooperia* spp., and *Trichostrongylus axei* [2,3]. In commercial grazing operations, calves may be exposed to GINs under natural conditions due to pasture contamination by infected herd mates or cattle who had previously grazed the pasture. Indirect negative effects of GIN infection result from the host’s response to infection, and direct negative effects result from the parasites’ dependance on host nutrients and physical damage to host tissues. Infection with GINs also results in parasite-induced hormonal changes, leading to reduced appetite [4]. The adverse effects caused by GIN infection most commonly manifest subclinically, with significant production losses as the only indication of GIN infection [5].

The most common management practice in stocker cattle to minimize losses attributable to GINs is the administration of anthelmintics to the entire population or group of cattle on arrival [3]. According to National Animal Health Monitoring System data, 73.2% of all operations dewormed weaned stocker calves at least occasionally [6]. In the East region of the US, nearly 80% of operations dewormed weaned stocker calves [6]. Most operations view the veterinarian as a very important source of information that influences their deworming practices [6]. Unfortunately, published data from well-designed commercial field trials evaluating deworming practices in stocker cattle are limited in this region.

There are currently three classes of anthelmintics approved for use in cattle in the US: benzimidazoles introduced in the 1960s, imidazothiazoles introduced in the 1970s, and *avermectin/milbemycins* (macrocyclic lactones) introduced in the 1980s [5]. Based on recent case reports and unpublished studies, anthelmintic resistance (AR) is a growing concern in cattle populations in the US [5]. Because of current reports on AR and the potential development of resistance, parasitologists and veterinarians have suggested and/or recommended management practices that can aid in slowing the development of resistance. Current recommendations include administering combination treatment and practicing refugia-based programs [5,7]. Combination treatment as it pertains to internal parasite control refers to administering two or more dewormers from different classes of drugs with different mechanisms of action [5]. Refugia is defined as the proportion of a parasite population not exposed to anthelmintic treatments and thus contributes susceptible alleles to the next generation [8]. The intended purpose of maintaining refugia is to dilute the potential resistance mechanism of the parasites that pass into the environment and reproduce. Selective nontreatment of a portion of the cattle population is one tactic proposed to reduce anthelmintic selection pressure and maintain a susceptible population of parasites. Pairing anthelmintic combinations with the implementation of refugia-based programs has proven effective in reducing the rate of AR in sheep populations [9].

Field data in cattle populations are limited. Kipp and colleagues assessed the effects of a refugia-based program on performance and fecal egg counts (FECs) in naturally infected beef calves concurrently administered anthelmintic combinations [10]. In that study, there were no significant differences in average body weight or average daily gain (ADG) when 10% of the animals in a group were selectively left untreated compared to whole-herd treatment, indicating no detrimental effect by leaving a proportion of cattle untreated. While not statistically different (*p* = 0.53), the means for body weight gain over the 131-day grazing season were 74.15 kg and 69.66 kg for groups receiving whole-herd treatment and the refugia (untreated) group, respectively [10]. Large-scale data from commercial field trials are needed to further elucidate the potential effects of different anthelmintics implemented concurrently with refugia-based programs.

Of the approved anthelmintics available for use in cattle in the US, only one is an extended-release formulation. Extended-release eprinomectin injectable is labeled for the treatment and control of internal and external parasites for cattle on pasture with persistent effectiveness. The duration of persistent effectiveness closely parallels common grazing periods (100 to 150 days) in many stocker systems. The duration of persistent effectiveness of conventional avermectins/milbemycins is dependent on product and parasite species.

A large-scale field trial conducted in Western Canada demonstrated significant increases in weight gain and ADG in cattle administered extended-release eprinomectin injectable compared to topical ivermectin [11]. To the authors’ knowledge, there are no peer-reviewed, published field trials evaluating the effects of different anthelmintics with the incorporation of refugia in a commercial stocker operation in the Mid-Atlantic region.

Based on previous research and current understandings, the authors hypothesized that extended-release eprinomectin injectable would improve ADG and decrease fecal egg shedding as measured by FEC compared to an anthelmintic with a shorter duration of activity when implemented in a commercial stocker cattle population with refugia. Thus, our objective was to compare the effects of extended-release eprinomectin injectable and doramectin injectable on growth and fecal shedding of trichostrongyle-type species and other non-trichostrongyle parasite species in market-derived stocker calves in the Mid-Atlantic region naturally exposed to GIN with the inclusion of a refugia-based program.

## 2. Materials and Methods

The primary objective of this study was to compare the effects of extended-release eprinomectin injectable and doramectin injectable on growth and fecal shedding in market-derived stocker cattle in the Mid-Atlantic region naturally exposed to GIN and with the inclusion of a refugia-based program. This study also presented an opportunity to assess variation in animal performance between different pastures within the same commercial operation under similar management conditions.

### 2.1. Experimental Design

This study was designed with individual animals as experimental and observational unit and pasture as the blocking factor. Sample size calculations were performed a priori to determine the number of animals (*n* = 474 per treatment) needed to detect a significant difference (*p* ≤ 0.05) in ADG of 0.045 kg/day during study days 0 to 105 between the two experimental groups with statistical power of 80%. Randomization and assignment to experimental group was performed by a blinded statistician. Animals not selected for refugia at arrival were stratified by arrival body weight and castration (or not) at time of arrival and randomized to one of two experimental groups (ERE or DOR) within each pasture. Individual animals selectively not treated with anthelmintics at arrival processing were not assigned to an experimental treatment group, thus serving as refugia (REF) for the duration of the grazing period [5].

### 2.2. Population

All study animals eligible for enrollment were procured from local livestock markets by a single commercial stocker operation and entered a 3-week backgrounding phase. Both bulls and steers were purchased over a 3-day period to quickly achieve the number of animals needed to execute the trial. On arrival (day 21) at the study site, animals were processed according to the operation’s health protocols. Intact bulls were castrated via surgical castration per protocol established by the attending veterinarian. Per standard protocol, animals received 22.5% oxfendazole oral suspension (Synanthic^®^; Boehringer Ingelheim Animal Health USA Inc., Duluth, GA, USA) at the labeled dose of 2.05 mg/lb (4.5 mg/kg). Every twentieth animal through the chute at arrival processing was selectively not treated with 22.5% oxfendazole oral suspension to serve as a source of refugia (REF). All steers observed to be visibly healthy following the 3-week backgrounding period were considered eligible for enrollment. At time of enrollment (day 0), all animals received the following: 5-way modified live respiratory vaccine (Pyramid^®^ 5; Boehringer Ingelheim Animal Health USA Inc., Duluth, GA, USA), autogenous respiratory bacterin (Autogenous Respiratory Bacterin; Cambridge Technologies, Worthington, MN, USA), autogenous pinkeye bacterin (Autogenous Pinkeye Bacterin; Cambridge Technologies, Worthington, MN, USA), one insecticide cattle ear tag (Corathon^®^; Elanco, Greenfield, IN, USA), and a 7-way clostridial bacterin-toxoid (Alpha™ 7; Boehringer Ingelheim Animal Health USA Inc., Duluth, GA, USA). In addition, steers assigned to one of the two treatment groups (ERE and DOR) received their respective treatment. On day 105 of the study, all animals (REF included) were administered 22.5% oxfendazole oral suspension (Synanthic^®^; Boehringer Ingelheim Animal Health USA Inc., Duluth, GA, USA) (2.05 mg/lb (4.5 mg/kg)), pour-on insecticide (Clean-Up™ II Pour-On Insecticide (Pyrethroid) with IGR; Elanco, Greenfield, IN, USA) (3 mL/100 lb (3 mL/45.5 kg)), and a trenbolone acetate (40 mg) and estradiol (8 mg) implant (Revalor^®^ IS; Merck Animal Health, Madison, NJ, USA). Excluding the anthelmintic treatments of interest, all health and management practices were standardized across the study population. All participants were challenged with natural exposure to GINs.

### 2.3. Experimental Treatments

Experimental treatments were administered by a licensed veterinarian according to individual animal body weight at the time of treatment (day 0). Animals in the ERE group (471 animals) received an injection of extended-release eprinomectin (LongRange^®^; Boehringer Ingelheim Animal Health USA Inc., Duluth, GA, USA) subcutaneously at a dosage of 0.45 mg/lb (1.0 mg/kg). Animals in the DOR group (477 animals) received an injection of doramectin (Dectomax^®^; Zoetis Inc., Parsippany-Troy Hills, NJ, USA) subcutaneously at a dosage of 0.091 mg/lb (0.20 mg/kg). Untreated animals (REF, *n* = 47) serving as a source of refugia did not receive experimental treatment on day 0 and were distributed evenly throughout the population to achieve 5% refugia across each of the six pastures. All animals (ERE, DOR, and REF) were commingled on respective pastures for the duration of the study, 130 days following experimental treatment administration. In the final analysis, 9 of the 471 enrolled animals in the ERE group were excluded, 4 due to mortality and 5 with incomplete individual body weight data, and 11 of the 466 animals enrolled in the DOR group were excluded, 4 due to mortality and 7 with incomplete individual body weight data.

### 2.4. Nutrition

Pastures accessed for grazing in this study were managed by a commercial stocker operation to produce yearling cattle developed on grass and supplied to commercial feedyards. In total, six pastures were assigned for use in this study by the operation. The assigned pastures comprised a native forage base typical for the Mid-Atlantic region consisting of fescue, bluegrass, white clover, and orchardgrass, and equipped with free choice open watering tanks. The animal stocking rate on pasture was two calves/acre, which was determined by historical stocking rates and forage growth. In the previous two years (2021 and 2022), stocker cattle grazed the same respective pastures at a stocking rate of 1.5 to 2 calves/acre following the administration of an endectocide and benzimidazole dewormer. In previous years, animals received fenbendazole in the ration late in the grazing period at the start of the supplementation program. On day 105, all animals were supplemented with a mixed ration targeting intake of 1% of body weight on a dry matter basis. The base ingredients for mixed ration were corn silage, cracked corn, triticale, and dried distiller grains (53 Mcals/cwt). A hand-fed stocker mineral (Ironsides Stocker Advantage Mineral MON1200; Hubbard Feeds, Mankato, MN, USA) was provided daily.

### 2.5. Outcomes

The primary response variables of interest included ADG and overall body weight gain as indicators of performance due to infection with GINs. Additional response variables of interest were FEC to determine fecal shedding of trichostrongyle-type species in eggs per gram (EPG) and the presence of other (non-trichostrongyle) parasite species. Larval culture of the feces (coproculture) was performed to identify and quantify genus/species of trichostrongyle-type eggs. All response variables of interest were collected and reported on days 0, 105, and 130 to parallel the critical end points for key measurements in this commercial production system.

Individual body weight was collected chute side on days 0, 105, and 130 to evaluate performance. Experienced caretakers employed by the operation were responsible for daily monitoring of animals and were blinded to experimental treatment of individual animals for the duration of the study. Animals were defined as “sick” based on subjective criteria established by the operation including depression, gauntness, nasal discharge, coughing, ocular lesions, and increased respiratory effort. Treatment interventions were recorded using commercially available cattle management software (Performance Livestock Analytics; Ames, IA, USA).

Fecal samples were collected per rectum for FEC of trichostrongyle-type species on days 0, 105, and 130. Approximately 10% of each group was chosen for fecal sampling by selecting the animals with the lowest numbered unique identification tag in each experimental treatment group (ERE and DOR). Fecal samples were collected from these same individual animals on days 0, 105, and 130. Fecal samples from all untreated animals (REF) were collected at each time point. All fecal samples were labeled according to individual ID and shipped with freezer packs overnight to a third-party veterinary diagnostic laboratory (Parasitology Diagnostic Laboratory, Texas A&M University; College Station, TX, USA). Fecal egg counts were performed using the Mini-FLOTAC technique. Eggs per gram were reported for trichostrongyle-type species and identified the presence of other non-trichostrongyle-type parasite species (*Eimeria* spp., *Strongyloides* spp., *Moniezia* spp., *Trichuris* spp., *Nematodirus* spp., and *Capillaria* spp.). In addition, coproculture was performed by pooling fecal samples from each experimental group (ERE or DOR) and REF animals by pasture to determine parasite species, i.e., a coproculture was performed on three pools (ERE, DOR and REF), from each pasture, at each time point (days 0, 105, and 130). Ten grams of each individual fecal sample with 10 EPG or more were homogenized. Vermiculite was added until reaching the desired consistency (roughly that of playdough). The mixture was wrapped in cheese cloth and suspended in an incubation chamber for 14 days. After incubation, the larvae were collected via Baermann apparatus and identified. Between 150 and 200 larvae were counted and identified from each pooled coproculture, and the percentages of each genus observed were reported. If under 150 larvae were recovered, all larvae were counted and identified. Laboratory personnel completing fecal egg counts and coprocultures were blinded to treatment.

All study data were organized into a spreadsheet program (Microsoft® 365 Excel; Microsoft Corporation, Redmond, WA, USA) and verified for statistical analyses.

### 2.6. Statistical Methods

Statistical analyses were performed based on the primary objective and study design structure defined a priori in the protocol and using a dataset with treatment groups masked. Data provided in spreadsheet files were reformatted for analyses in SAS (version 9.4) (SAS 9.4; SAS Inst., Inc., Cary, NC, USA). Linear mixed models were used for all data analyses (Proc GLIMMIX SAS 9.4) with null hypotheses that ERE and DOR means were equal, i.e., the a priori contrast of interest. Individual animals were the unit of replication (i.e., experimental unit). Random intercepts were used to account for the lack of independence among animals within sex and within pasture. Animals selected for refugia (REF) are not in the statistical analysis as REF was not an experimental treatment group. Fecal egg count data were analyzed with a negative binomial distribution and log link, and the probability of shedding other non-trichostrongyle-type parasite species (described above) based on dichotomous presence/absence for each fecal was analyzed with a binomial distribution and logit link; both were back-transformed for displaying results. Normal distributions (identity link) were used for weight and weight gain models. A secondary analysis was performed to assess potential differences in ADG among pastures where pasture, castration at arrival, treatment and their interaction terms were all fitted as fixed effects. For all results, model-adjusted means (at the original scale) and corresponding standard errors (SEM) are reported. The results are interpreted with an alpha-level of 0.05 with a tendency considered for a *p* value of 0.05 < *p* ≤ 0.10 as per Auchard et al. [12].

## 3. Results

Of the 995 animals enrolled on day 0 (May 2023), data were analyzed for 975 (ERE = 462, DOR = 466, REF = 47) at the conclusion of the study on day 130 (September 2023). Day 0 body weights ranged from 165.5 to 342.7 kg. Table 1 includes descriptive data on animals enrolled in the study. Table 2 summarizes the results of analyses of pre-treatment baseline data for all enrolled animals completing the trial period.

### 3.1. Animal Health (Descriptive Results)

Mortalities reported by study monitors in the ERE group included two attributable to bovine respiratory disease (BRD) and two classified as pen dead. In the DOR group, three deaths were attributed to BRD and one pen dead. Classification of “pen dead” was assigned for an animal found suddenly deceased with no apparent cause of mortality. There were no mortalities reported in the untreated animals (REF). Post-mortem necropsies were not performed to confirm the cause of mortality. In total, 28 treatments were administered for subjective clinical signs of BRD. Thirteen treatments were administered to animals in the ERE group, fourteen to animals in the DOR group, and one treatment for BRD was administered in the untreated animals (REF). A total of ten treatments were administered due to clinical signs of infectious bovine keratoconjunctivitis, one in the ERE group and nine in the DOR group. There were no reported adverse events due to experimental treatment in the ERE or DOR groups.

### 3.2. Performance (Body Weight Gain)

Individual body weight data recorded at days 0, 105, and 130 were used to assess ADG and total body weight gain during the study period. There were no statistically significant differences in overall gain (day 0 to 130), in body weight (days 0, 105, or 130), or in ADG from day 105 to 130 (Table 3). However, the period of the primary hypothesized difference in ADG between the ERE and DOR groups was the period from day 0 to 105 (Table 3). From day 0 to 105, the mean ADG in the ERE group was 0.87 kg/day, which tended (*p* = 0.055) to be higher than the mean for the DOR group, 0.845 kg/day. Separate models demonstrated no evidence of an interaction between treatment and castration (or not) on arrival.

### 3.3. Fecal Results

A summary of results of fecal data analyses from 10% of the study population and all REF calves is presented in Table 4. Mean FEC was significantly lower in the ERE group on day 105 and 130 when compared to the DOR group. On day 105, mean FEC in the ERE group was 46.45 EPG compared to 155.30 EPG in the DOR group (*p* < 0.01). Subsequently, on day 130, the mean FEC was also significantly lower (*p* = 0.02) in the ERE group (9.65 EPG) compared to the DOR group (22.51 EPG). Mean FEC in the REF calves remained similar from day 0 (133.31 EPG) to day 105 (133.58 EPG) indicating fecal shedding of eggs. On day 105, the DOR group had a significantly greater percentage of animals with other non-trichostrongyle parasites (49.06%) compared to the ERE group (26.51%) (*p* = 0.03). Larvae were not recovered via coproculture from the ERE and DOR groups on day 0. Of the larvae recovered from the REF calves on day 0, 82.3% were *Cooperia,* 0.7% *Haemonchus*, 16% *Ostertagia*, 0.4% *Oesophagostomum*, and 0.6% *Trichostrongylus.* Larvae were recovered from the ERE and DOR groups and refugia animals on days 105 and 130. A summary of the averages of larvae recovered on days 0, 105, and 130 is presented in Table 5.

### 3.4. Variation in ADG by Pasture

Secondary analyses were performed to evaluate potential differences among pastures in three measures of ADG (days 0 to 105, 105 to 130, and 0 to 130). None of these analyses had significant evidence of interactions among pasture, treatment, and castration (or not) (*p* values > 0.1;), but there were significant differences by pasture for all three ADG timepoints after controlling for the effects of treatment and arrival sex status (*p* values < 0.01). Differences among pastures in ADG are shown in Table 6.

## 4. Discussion

The primary objective of this study was to evaluate the effects of extended-release eprinomectin injectable and doramectin injectable on performance outcomes and fecal variables in market-derived stocker calves with the inclusion of refugia in the Mid-Atlantic region. To the authors’ knowledge, there are limited studies in the published literature addressing this objective in stocker cattle in the Mid-Atlantic regions. Parasitologists and industry stakeholders have proposed maintaining refugia in cattle populations and combination treatment to delay AR and preserve the efficacy of current anthelmintics, yet limited large-scale field data are available [5]. Including a long-acting formulation in the anthelmintic treatment regimen centered around combination treatment and refugia management may be a highly effective approach [7].

Extended-release eprinomectin injectable is the only extended-release anthelmintic approved for cattle in the US. Previous research has demonstrated persistent effectiveness and production benefits following administration of extended-release eprinomectin injectable to growing animals on pasture [11,13,14,15,16,17,18]. Similarly to previous research, in the current study, there was a tendency towards a difference in ADG between the ERE group and DOR group during the grazing period (day 0 to 105). There were no statistically significant differences in performance outcomes observed at any other time points.

The commingling of experimental groups also may have served as a limitation to identifying differences in performance variables between the two interventions. In a previous study, Rademacher and colleagues surmised that fecal shedding may differ between experimental treatment groups (extended-release eprinomectin injectable and topical avermectin) during the grazing period [11]. The current study confirms significant differences in fecal shedding between the 10% of the population sampled in each experimental group (ERE and DOR) at the end of the grazing period. Mean FEC and percentage of animals with other parasites were not statistically different at enrollment, possibly due to the administration of 22.5% oxfendazole oral suspension at arrival (21 days prior to start of study), which was in accordance with the operation’s standard procedures. In addition, administration of a benzimidazole prior to experimental treatment ensured minimal differences in parasite burden between the two experimental groups on day 0. Establishing a similar parasite burden prior to experimental treatment administration enabled any significant differences in response variables to be attributed to the experimental treatment [18]. Potential differences in fecal shedding were expected as the duration of persistent effectiveness for extended-release eprinomectin injectable is longer than doramectin injectable per each product’s label.

Regarding parasitological outcomes, a limitation of the current study was the selection of study animals within experimental treatment groups for fecal collection. The non-random selection of a subset of animals (10%) with the lowest unique identification tag for fecal collection per rectum occurred following randomization of all animals to each respective experimental treatment group. Although selecting the animals with the lowest unique identification tag could result in a non-representative sample of the population as a whole, the selection approach was the same across treatment groups (ERE and DOR) and thus unbiased with respect to the hypotheses tested. Nonetheless, the fecal shedding outcomes must be interpreted with consideration that the results may not apply to the entire study population, but only the animals sampled. A second limitation regarding parasitological outcomes is the timing of fecal sampling. The study was designed to test a specific hypothesis while mirroring logistics and potential adoption in a commercial stocker operation. Additional fecal collection points to complete a FECRT (fecal egg count reduction test) in accordance with parasitologist recommendations for diagnosing anthelmintic resistance or testing product efficacy were not warranted or feasible [5]. The investigators appreciate the need for future clinical research aligning with these recommendations to better understand the significance of the parasite burdens observed and potential interactions; however, this was not within the scope of the current study objective.

Study animals became infected with GINs via natural challenge during the grazing period as indicated by an increase in mean FEC between day 0 and 105. The authors speculate that animals selectively not treated (REF) at arrival and enrollment (day 0 FEC: 133.31 EPG) may have contributed to pasture contamination leading to reinfection in the experimental groups. It is also possible the source of parasite infection during the trial period could have resulted from previous pasture contamination on the farm or established GIN infection from the farm of origin. Nontreated animals harboring GINs serve to maintain a population of parasites in the host, termed “infrapopulation”, not exposed to anthelmintics; therefore, propagating susceptible alleles to future generations [8]. Previous researchers evaluated the effects of implementing refugia via selective nontreatment and combination treatment in a parasite control program in stocker cattle [10]. The investigators did not identify significant differences in ADG or BW when comparing whole herd treatment and inclusion of 10% refugia with the same anthelmintic combinations [10]. In the current study, anthelmintic treatment was withheld from 5% of the study animals. This percentage was a compromise between study investigators and the commercial operation. Oral oxfendazole was administered on day 105 to all study animals, including REF calves, to mitigate the potential negative impacts of internal parasitism during the supplementation phase (day 105 to 130) prior to movement of animals to the feedyard.

Although the REF data were not analyzed statistically, the overall gain (day 0 to 130) in the ERE and DOR groups compared to the REF results could be a potential indication of performance loss in the untreated animals (Table 3). To mitigate the potential for performance loss in untreated animals, researchers have proposed leaving the heaviest animals in the herd untreated [5]. The approach used in the current study to select animals for refugia was considered the most practical method for a commercial stocker operation. The results observed on day 105 of the study provide no evidence to suggest that subsequent treatment with a benzimidazole near the end of the grazing season resulted in complete compensatory gain. Potential performance losses associated with maintaining refugia must be considered at a systems level given the complex interactions observed in stocker cattle systems [19]. Short-term production and economic losses may be incurred while identifying leverage points to preserve anthelmintic efficacy. Significant investments must be made to conduct long-term clinical field trials to better understand the complexity of these interactions and potential unintended consequences.

Documenting the significant differences in ADG by pasture was a unique finding in this study (Table 6). Historical pasture management and stocking rates were similar across the six pastures represented in the study. Pastures were geographically located within approximately a 2-mile (3.2 km) radius. Supplementation and management of study animals were standardized across the six pastures for the duration of the study. Furthermore, of the six cattle sources used to procure cattle, all were represented across the six different pasture groups. Lastly, intact males at arrival were allocated to each of the six pasture groups along with steer cohorts via randomization to each experimental treatment group. Nonetheless, statistically significant, and relatively important differences, in ADG by pasture were observed after controlling for experimental treatment and arrival sex. Assigning experimental groups to whole pastures would have been a significant oversight in the experimental design given the limited number of pastures and significant variability among pastures. Hence, the commingled experimental design with individual animals serving as the experimental unit was critical for this study and has implications for future studies.

## 5. Conclusions

In conclusion, calves administered extended-release eprinomectin tended to have a higher average daily gain during the 105-day grazing period compared to calves administered doramectin injectable. Additionally, in a subset of the study population, calves administered extended-release eprinomectin shed fewer parasite eggs on day 105 of the grazing period as compared to calves administered doramectin injectable. Future research is needed to gain a deeper understanding of parasite control practices, anthelmintic resistance and efficacy of dewormers in stocker operations. Specifically, long-duration clinical field trials evaluating the implications of refugia-based parasite control programs are warranted.

## Figures and Tables

**Table 1:** Data collected at enrollment (day 0) for field trial evaluating different anthelmintic treatments and refugia (REF) in stocker cattle. ERE were given extended-release eprinomectin injectable and DOR were given doramectin injectable.

	ERE		DOR		REF		Overall		
Arrival Sex	N	Mean BW *, kg	N	Mean BW, kg	N	Mean BW, kg	N	Mean BW, kg	Range BW, kg
Bull **	94	237.3	96	238.28	12	240.45	202	237.95	165–311
Steer	377	244.97	381	244.62	35	243.92	793	244.75	175–343
Overall	471	243.45	477	243.34	47	243.04	995	243.38	165–343

* BW: Body weight; ** Castrated on day-21 (on-arrival).

**Table 2:** Model-adjusted * means and standard errors (SEMs) by treatment group for baseline data for experimental groups. ERE were given extended-release eprinomectin injectable and DOR were given doramectin injectable. REF indicates untreated animals serving as a source of refugia.

	ERE Treatment Mean and SEM		DOR Treatment Mean and SEM		*p* Value for Treatment	REF Mean and SEM	
Arrival (Day 21) Body weight, kg	220.72	6.93	221.44	6.92	0.68	218.49	8.71
Day 0, Body weight, kg	236.01	4.32	236.17	4.32	0.92	235.34	5.22
Day 0, eggs per gram	0.18	0.39	0.02	0.06	0.28	133.31	51.64
** Day 0 animals with other parasites, %	57.43%	8.78%	63.05%	8.72%	0.59	73.27%	7.21%

* account for the lack of independence among animals within sex and within pasture. ** presence/absence of non-trichostrongyle-type parasite species.

**Table 3:** Summary of performance data from study population evaluating two different anthelmintic treatments with the inclusion of refugia (REF). Model-adjusted * means and standard errors (SEM) by treatment group (ERE: extended-release eprinomectin injectable; DOR: doramectin injectable).

	ERE Treatment Mean and SEM		DOR Treatment Mean and SEM		*p* Value for Treatment	REF Mean and SEM	
Day 0 Body weight, kg	236.01	4.32	236.17	4.32	0.92	235.34	5.22
Day 105 Body weight, kg	328.26	5.37	325.21	5.37	0.16	321.75	7.08
Day 130 Body weight, kg	357.21	5.28	354.83	5.27	0.32	349.72	7.27
Day 0 to 105 ADG, kg	0.87	0.05	0.85	0.05	0.055	0.82	0.06
Day 105 to 130 ADG, kg	1.16	0.09	1.18	0.09	0.62	1.13	0.12
Day 0 to 130 ADG, kg	0.93	0.05	0.91	0.05	0.19	0.88	0.05
Day 0 to 130 Overall Gain, kg	120.60	5.95	118.34	6.01	0.19	114.38	7.13

* models account for the lack of independence among animals within sex and within pasture.

**Table 4:** Results of data analyses for fecal egg count (eggs per gram (EPG)) from approximately 10% of the study population and percentage of animals with other (non-trichostrongyle) parasite species between two experimental treatments (ERE: extended-release eprinomectin injectable; DOR: doramectin injectable) and all refugia (REF) calves. Model-adjusted * means and standard errors (SEMs) by treatment group with inclusion of refugia (REF) calves.

	ERE Treatment Mean and SEM		DOR Treatment Mean and SEM		*p* Value for Treatment	REF Mean and SEM	
Day 0, EPG	0.28	0.15	0.07	0.05	0.03	133.31	51.64
Day 105, EPG	46.45	16.82	155.30	52.25	<0.01	133.58	43.63
Day 130, EPG	9.65	4.12	22.51	9.78	0.02	12.41	4.94
** Day 0 animals with other parasites, %	58.15%	8.55%	63.59%	8.47%	0.59	73.27%	7.21%
** Day 105 animals with other parasites, %	26.51%	7.70%	49.06%	9.26%	0.03	39.05%	8.43%
** Day 130 animals with other parasites, %	40.52%	8.70%	54.62%	8.96%	0.18	46.11%	8.48%

* models account for the lack of independence among animals within gender and within pasture. ** presence/absence of non-trichostronglye-type parasite species.

**Table 5:** Summary of pooled coproculture results from a field trial evaluating the effects of two anthelmintic treatments commingled with untreated refugia (REF) calves at three timepoints (Day 0, 105, and 130). (ERE: extended-release eprinomectin injectable; DOR: doramectin injectable) “-“ indicates no larvae were recovered.

		ERE Treatment			DOR Treatment			REF		
		Total Larvae Count *	Larvae Percentage	Average Larvae **	Total Larvae Count *	Larvae Percentage	Average Larvae **	Total Larvae Count *	Larvae Percentage	Average Larvae **
Day 0	*Cooperia*	-	-	-	-	-	-	442	82.3%	78.7
	*Haemonchus*	-	-	-	-	-	-	4	0.7%	0.7
	*Ostertagia*	-	-	-	-	-	-	86	16%	14.3
	*Oesophagostomum*	-	-	-	-	-	-	2	0.4%	0.3
	*Trichostrongylus*	-	-	-	-	-	-	3	0.6%	0.5
Day 105	*Cooperia*	445	74.2%	74.2	719	87.5%	119.8	640	75.5%	106.7
	*Haemonchus*	2	0.3%	0.33	-	-	-	-	-	-
	*Ostertagia*	153	25.5%	25.5	103	12.5%	17.2	204	24.1%	34
	*Oesophagostomum*	-	-	-	-	-	-	-	-	-
	*Trichostrongylus*	-	-	-	-	-	-	4	0.5%	0.7
Day 130	*Cooperia*	223	85.4%	37.2	260	90%	43.3	222	84.1%	37
	*Haemonchus*	-	-	-	-	-	-	-	-	-
	*Ostertagia*	38	14.6%	6.3	29	10%	4.8	42	15.9%	7
	*Oesophagostomum*	-	-	-	-	-	-	-	-	-
	*Trichostrongylus*	-	-	-	-	-	-	-	-	-

* represents the total number (sum) of larvae reported for all coproculture pools by treatment group (ERE and DOR) and REF animals. ** represents the average number of larvae reported per coproculture pool by treatment group (ERE and DOR) and REF animals.

**Table 6:** Average daily gain (ADG) by pasture from a field trial evaluating the effects of different anthelmintic treatments (ERE: extended-release eprinomectin injectable; DOR: doramectin injectable) commingled with untreated cattle. Model-adjusted means and standard errors (SEMs) presented by pasture.

	ADG Days 0 to 105, kg Mean and SEM		ADG Days 105 to 130, kg Mean and SEM		ADG Days 0 to 130, kg Mean and SEM	
Pasture 1	0.89 ^a^	0.05	1.22 ^a^	0.12	0.95 ^ab^	0.05
Pasture 2	1.03 ^a^	0.05	1.39 ^a^	0.12	1.10 ^a^	0.05
Pasture 3	0.91 ^a^	0.05	1.50 ^a^	0.12	1.02 ^ab^	0.05
Pasture 4	0.96 ^a^	0.03	0.82 ^b^	0.07	0.94 ^b^	0.03
Pasture 5	0.66 ^b^	0.03	0.82 ^b^	0.07	0.69 ^c^	0.02
Pasture 6	0.61 ^b^	0.05	1.20 ^ab^	0.12	0.72 ^c^	0.05

Within columns, means without a common superscript differ (*p* value < 0.05).

## Data Availability

The raw data supporting the conclusions of this article will be made available by the authors on request.
